# Severe cytomegalovirus encephalitis in an immunocompetent man: a case report

**DOI:** 10.1093/bjrcr/uaaf050

**Published:** 2025-10-10

**Authors:** Fatima Chait, Nourrelhouda Bahlouli, Adam Sqalli Houssaini, Ihssane Lahlou, Meryem Edderai, Jamal El Fenni

**Affiliations:** Radiology Department, Military Teaching Hospital Mohammed V, Mohammed V University, Rabat 15000, Morocco; Radiology Department, Military Teaching Hospital Mohammed V, Mohammed V University, Rabat 15000, Morocco; Radiology Department, Military Teaching Hospital Mohammed V, Mohammed V University, Rabat 15000, Morocco; Radiology Department, Military Teaching Hospital Mohammed V, Mohammed V University, Rabat 15000, Morocco; Radiology Department, Military Teaching Hospital Mohammed V, Mohammed V University, Rabat 15000, Morocco; Radiology Department, Military Teaching Hospital Mohammed V, Mohammed V University, Rabat 15000, Morocco

**Keywords:** cytomegalovirus, encephalitis, immunocompetent

## Abstract

Cytomegalovirus (CMV) is a viral infection that is generally considered benign in immunocompetent patients; however, it can be life-threatening in immunocompromised patients. We present the case of a 46-year-old patient with severe primary CMV encephalitis. The patient presented to the hospital emergency department with impaired alertness and speech. A brain scan and initial lumbar puncture (LP) were normal. However, the patient’s state of consciousness deteriorated rapidly with the onset of seizures, prompting a brain MRI scan, which revealed lesions suggestive of encephalitis. Polymerase Chain Reaction (PCR) detection of cytomegalovirus was confirmed on a second lumbar puncture. Following the confirmation of the diagnosis, the patient was commenced on appropriate antiviral treatment, which resulted in a favourable outcome. Our aim is to report on the clinical manifestations and the contribution of imaging to the diagnosis of CMV encephalitis in immunocompetent persons.

## Introduction

Cytomegalovirus (CMV) is a herpesvirus with a global prevalence ranging from 45% to 100%. Primary infection acquired during adolescence is often asymptomatic or may present as mononucleosis-like or influenza-like illness in immunocompetent adults.^1^ In healthy adults, the virus enters a lifelong latent phase within peripheral monocytes and CD34+ myeloid progenitor cells. However, it can reactivate in immunocompromised patients, leading to severe, life-threatening disease. Moreover, high mortality and morbidity rates have been reported in critically ill immunocompetent patients.^2^ Severe CMV infection remains rare in previously healthy adults.^3^ Delayed diagnosis in immunocompetent patients can result in serious complications. This case report describes a severe primary CMV encephalitis in an immunocompetent patient.

## Case report

The patient was 46 years of age and had no previous medical history. One week before admission, the patient presented with subacute alterations in alertness and speech disturbance, as well as 2 prolonged generalized tonic-clonic seizures (lasting 15 minutes each). An initial CT scan of the brain revealed no abnormalities.

However, as his state of consciousness deteriorated, he was transferred to our hospital for further treatment. On admission, the patient exhibited altered consciousness with a Glasgow score of 8, with no sensory-motor deficits or other associated signs.

The results of the laboratory work-up were unremarkable, with a white blood cell count of 13,000/mm³, a negative inflammatory work-up, PCR tests for HIV, syphilis, and hepatitis were performed and all were negative. Additionally, primary immune function tests were conducted and yielded satisfactory results. Therefore, an immunodeficiency was ruled out. The metabolic panel was normal. The biochemical and cytobacteriological examination of the cerebrospinal fluid (CSF) revealed a proteinorachy of 0.45 g/L, normal glycorachy, 2 leukocytes/mm³ and no haematocytes. Furthermore, the toxicology screening was unremarkable.

**Figure 1.**
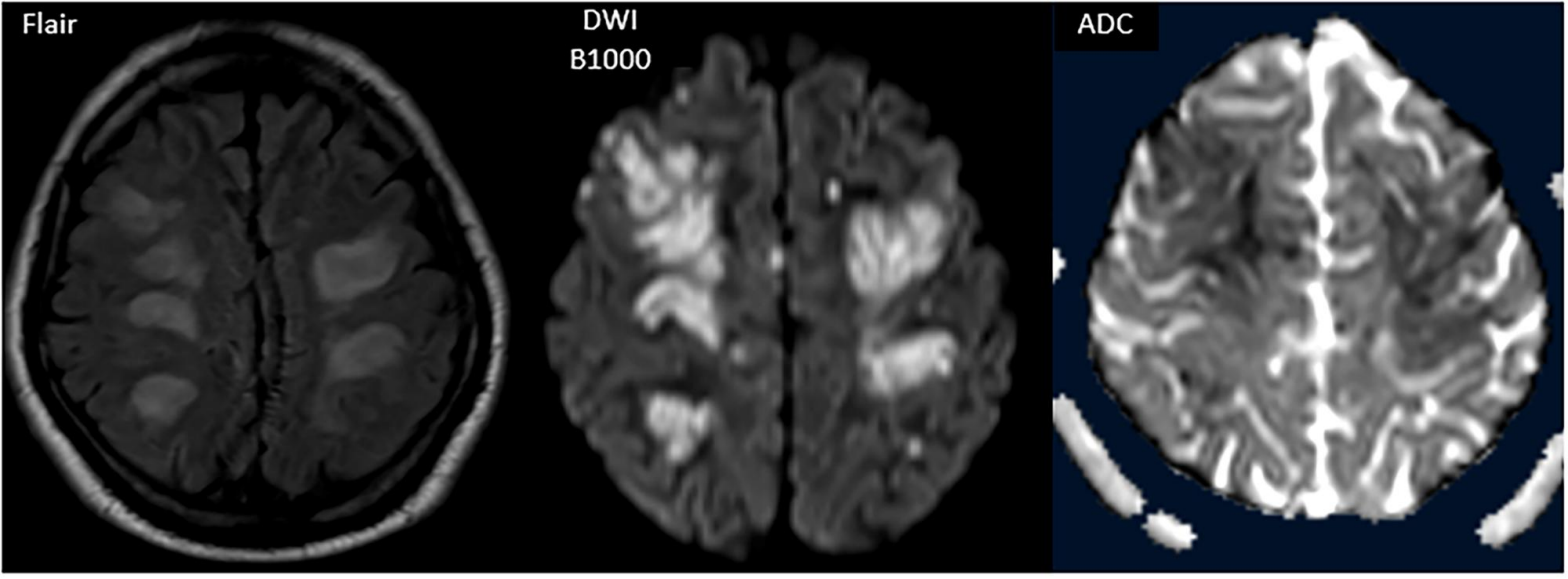
T2-weighted FLAIR and diffusion-weighted imaging (b = 1000 s/mm^2^) with apparent diffusion coefficient (ADC) mapping, passing through the convexity white matter. Signal abnormalities are seen in the white matter as FLAIR hyperintensity with corresponding diffusion restriction, which is notably limited to the periphery of the larger lesions as confirmed on the ADC map.

**Figure 2.**
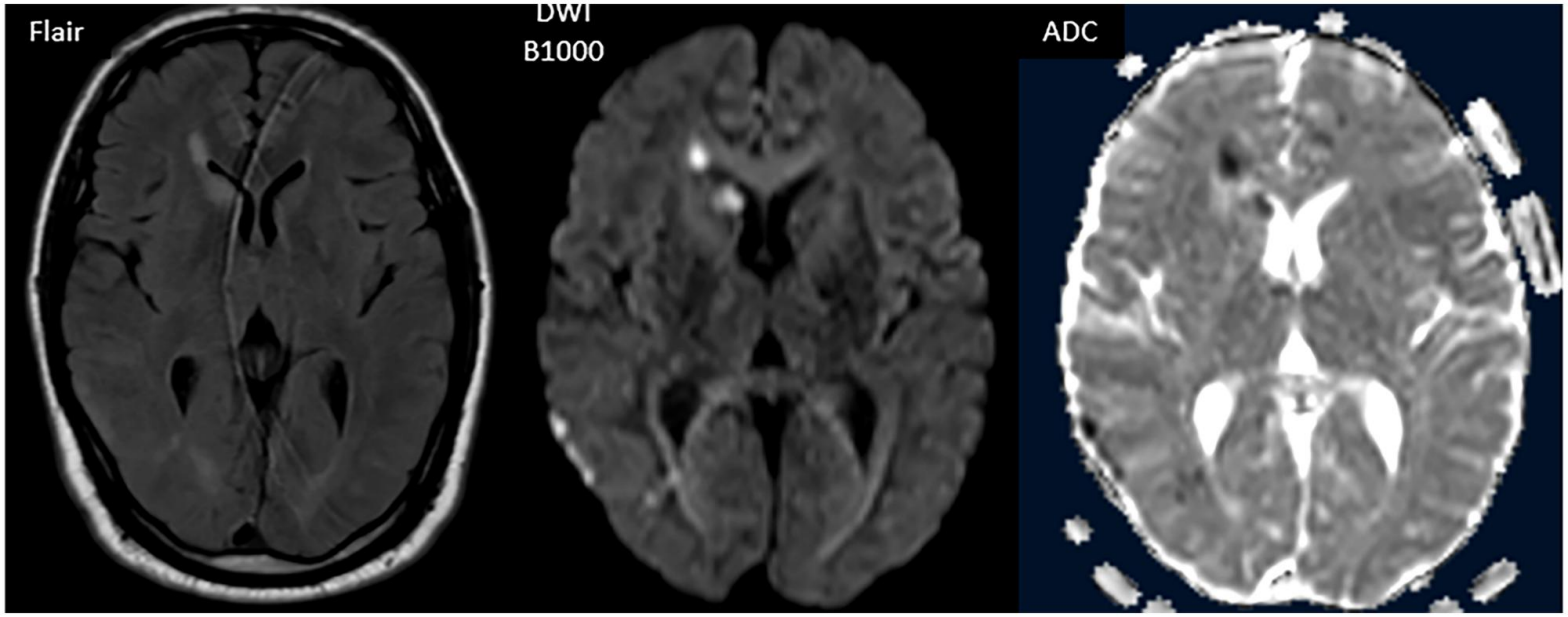
T2-weighted FLAIR, diffusion (B1000) imaging with apparent diffusion coefficient (ADC) mapping, passing through the thalamus and caudate nuclei, revealing FLAIR hyperintensities and diffusion restriction in areas lacking clear delineation, including the head of the caudate nucleus and periventricular white matter. Note: The image quality of the FLAIR sequences is somewhat limited due to patient confusion during the MRI acquisition.

In view of this clinical picture, a brain MRI was performed, revealing bilateral cortico-subcortical lesions with sulcal effacement, with a fronto-parietal and occipital predominance, as well as T2 and FLAIR hyperintensities in the periventricular white matter and the right caudate nucleus. These lesions were diffusion-restricted, with no enhancement after gadolinium injection and no signs of hydrocephalus, suggesting encephalitis lesions (Figures 1 and 2).

A multiplex PCR was also performed on the CSF, revealing the presence of CMV antigen; however, antigens for herpes simplex virus, Haemophilus influenzae, parechovirus, enterovirus, and varicella virus were absent.

Given the clinical picture of encephalopathy, the results of the MRI and the positive CMV PCR in the CSF, the diagnosis was that of CMV encephalitis, with no underlying immunosuppression. The patient received antiviral treatment with Ganciclovir (5 mg/kg/IV q12h), corticosteroids, and anti-convulsant therapy, leading to significant clinical improvement after one week. However, no follow-up MRI was performed.

## Discussion

Cytomegalovirus is a virus belonging to the Herpesviridae family. It is ubiquitous and infects nearly all individuals, with approximately 40% to 100% of healthy individuals showing positive serology (CMV antibodies).^1^ Primary infection is usually asymptomatic in immunocompetent hosts but may cause mild flu-like symptoms such as fever, pharyngitis, and lymphadenopathy. However, CMV is considered a significant cause of morbidity and mortality in immunocompromised patients, such as those with HIV/AIDS, individuals undergoing immunosuppressive therapy, and transplant recipients.

Cytomegalovirus, the most common human herpesvirus, possesses a linear double-stranded DNA genome encoding approximately 165 proteins. These proteins mimic and interact with human cellular proteins, contributing to the virus’s virulence and latency. Cytomegalovirus remains latent or exhibits low-level activity in monocytes, particularly dendritic cells, due to the immune response from CD8+ cytotoxic T lymphocytes and memory T cells. In immunocompetent individuals, asymptomatic viral shedding can be detected in saliva or urine; however, host cell-mediated immune responses prevent the development of overt CMV disease.^4^

Cytomegalovirus encephalitis predominantly occurs in immunocompromised patients, especially those with HIV/AIDS. Neurological involvement by CMV is uncommon, constituting less than 1% of CMV infections in this group.^5^ However, such infections are severe and can result in up to 100% mortality if untreated. In contrast, CMV encephalitis in immunocompetent individuals is exceedingly rare, with only a few cases reported.^6^

Neurological involvement can manifest in various ways, ranging from asymptomatic presentations to confusion, seizures, and cranial nerve palsies. Diagnosis is confirmed through viral PCR of the CSF. MRI typically reveals increased T2/FLAIR signal in white matter regions. In cases of concurrent ventriculitis, enhancement of the ependymal surface and hydrocephalus may also be observed.^7^

An unusual feature in this case was the widespread diffusion restriction observed on MRI. Diffusion restriction typically reflects cytotoxic edema caused by acute cellular injury. In the context of CMV encephalitis, this may indicate extensive viral-induced inflammation or ischemic injury to the white matter. Although rare, such findings have been reported and suggest a more severe disease course or involvement of both inflammatory and vascular mechanisms.^8^

Although CMV infection has been widely studied in immunocompromised populations, including transplant recipients and patients with advanced HIV infection,^9^^,^^10^ reports of CMV encephalitis in immunocompetent individuals remain exceptionally rare. A limited number of case reports describe central nervous system involvement in immunocompetent hosts, typically presenting with encephalitis, ventriculitis, or myelitis.^11^ These observations suggest that severe neurological complications can occasionally occur even in the absence of underlying immunosuppression, challenging the conventional understanding of CMV as an opportunistic pathogen. Our case contributes to this limited body of literature by emphasizing the diagnostic utility of MRI and CSF-PCR, and by illustrating the need to consider CMV in the differential diagnosis of encephalitis—even in immunocompetent patients presenting with nonspecific neurologic symptoms.

The main differential diagnoses included HIV encephalitis, progressive multifocal leukoencephalopathy, and CNS lymphoma, which typically occur in immunocompromised patients. As our patient was HIV-negative with no known immunodeficiency, these were considered unlikely. The diagnosis of CMV encephalitis in an immunocompetent host was confirmed by positive CMV PCR in the CSF.^12^

Treatment of CMV encephalitis typically involves antiviral therapy with ganciclovir, administered intravenously at a dose of 5 mg/kg every 12 hours for at least 21 days.^13^

It is important to note that antiviral treatment can be associated with side effects, notably hematological toxicity. However, in immunocompetent patients treated with ganciclovir, no serious side effects have been reported. In the absence of specific data for immunocompetent patients, treatment recommendations are often extrapolated from protocols used in immunocompromised patients.^14^

## Learning points

- Cytomegalovirus (CMV) is a rare cause of viral encephalitis in adults and is generally observed in patients with compromised immune systems.
- There are few documented cases in the literature of CMV encephalitis in immunocompetent patients.
- Radiological findings are not specific and the diagnosis is based on the detection of the virus by PCR in the CSF.
